# A cohort study to define the age-specific incidence and risk factors of *Shigella* diarrhoeal infections in Vietnamese children: a study protocol

**DOI:** 10.1186/1471-2458-14-1289

**Published:** 2014-12-17

**Authors:** Corinne N Thompson, Katherine L Anders, Le Thi Quynh Nhi, Ha Thanh Tuyen, Pham Van Minh, Le Thi Phuong Tu, Tran Do Hoang Nhu, Nguyen Thi Thanh Nhan, Tran Thi Thao Ly, Vu Thuy Duong, Lu Lan Vi, Nguyen Thi Van Thuy, Nguyen Trong Hieu, Nguyen Vinh Van Chau, James I Campbell, Guy Thwaites, Cameron Simmons, Stephen Baker

**Affiliations:** Wellcome Trust Major Overseas Programme, Oxford University Clinical Research Unit, Ho Chi Minh City, Vietnam; Centre for Tropical Medicine, Nuffield Department of Medicine, University of Oxford, Oxford, UK; The London School of Hygiene & Tropical Medicine, London, UK; Department of Epidemiology and Preventive Medicine, Monash University, Melbourne, Australia; Hospital for Tropical Disease, Ho Chi Minh City, Vietnam; Hung Vuong Hospital, Ho Chi Minh City, Vietnam; Department of Microbiology & Immunology, University of Melbourne, Melbourne, Australia

**Keywords:** Diarrhoea, *Shigella*, Active surveillance, Vietnam, Cohort

## Abstract

**Background:**

*Shigella* spp. are one of the most common causes of paediatric dysentery globally, responsible for a substantial proportion of diarrhoeal disease morbidity and mortality, particularly in industrialising regions. Alarming levels of antimicrobial resistance are now reported in *S. flexneri* and *S. sonnei*, hampering treatment options. Little is known, however, about the burden of infection and disease due to *Shigella* spp. in the community.

**Methods/Design:**

In order to estimate the incidence of this bacterial infection in the community in Ho Chi Minh City, Vietnam we have designed a longitudinal cohort to follow up approximately 700 children aged 12–60 months for two years with active and passive surveillance for diarrhoeal disease. Children will be seen at 6 month intervals for health checks where blood and stool samples will be collected. Families will also be contacted every two weeks for information on presence of diarrhoea in the child. Upon report of a diarrhoeal disease episode, study nurses will either travel to the family home to perform an evaluation or the family will attend a study hospital at a reduced cost, where a stool sample will also be collected. Case report forms collected at this time will detail information regarding disease history, risk factors and presence of disease in the household.

Outcomes will include (i) age-specific incidence of *Shigella* spp. and other agents of diarrhoeal disease in the community, (ii) risk factors for identified aetiologies, (iii) rates of seroconversion to a host of gastrointestinal pathogens in the first few years of life. Further work regarding the longitudinal immune response to a variety of *Shigella* antigens, host genetics and candidate vaccine/diagnostic proteins will also be conducted.

**Discussion:**

This is the largest longitudinal cohort with active surveillance designed specifically to investigate *Shigella* infection and disease. The study is strengthened by the active surveillance component, which will likely capture a substantial proportion of episodes not normally identified through passive or hospital-based surveillance. It is hoped that information from this study will aid in the design and implementation of *Shigella* vaccine trials in the future.

## Background

Diarrhoea remains a major cause of childhood morbidity and mortality globally [1, 2], with the vast majority of the 1.7 billion annual infections and 0.7 million deaths occurring in low and middle-income countries [3]. The four species of the Gram-negative bacterial genus *Shigella* (*S. flexneri, S. sonnei, S. boydii* and *S. dysenteriae*) are amongst the most common causes of dysenteric diarrhoea worldwide, with >164 million infections estimated annually in 1999 [4]. There is a growing necessity to prevent and control *Shigella* infections due to a dramatic emergence of highly resistant strains in not only southeast Asia [5–7], but in many other countries in both high and low income settings [8–16]. There are also extensive knowledge gaps regarding the distribution, incidence and exposure to *Shigella* spp., including a paucity of information on age-specific incidence, which is essential for assessing disease burden and evaluating the benefits of any future vaccines.

To address the outstanding epidemiological questions related to *Shigella* infections, we are conducting a longitudinal cohort study focusing on diarrhoeal disease with both routine and active surveillance components in Ho Chi Minh City (HCMC) in southern Vietnam. HCMC is a densely populated, rapidly urbanizing setting home to over 7.5 million people with a per-capita annual income of $1,220 [17]. The burden of *Shigella* infections is known to be substantial in children in Vietnam [5, 18]. A previous cohort study conducted in the early 2000s in the coastal province of Khanh Hoa in central Vietnam estimated an incidence rate of 490/100,000/year in children under the age of five years through passive surveillance of diarrhoeal cases [19]. Although several longitudinal, community-based cohorts in Southeast Asia have been established within the last two decades to investigate diarrhoeal disease, very few have included an active disease surveillance component [19–24]. Active surveillance is challenging for obvious reasons, including the substantial cost, intensive staff and resource needs, difficulty in ascertainment and confusion on what is considered to be an ‘episode’ of diarrhoea by parents or guardians.

The overall aim of this study is to describe the epidemiology of *Shigella* infections in HCMC to inform the development and introduction of *Shigella* vaccines. The primary objective of this study will be to define the age-specific incidence and exposure of *Shigella* infection in children ≤ 60 months of age in the community in HCMC. Secondary objectives include (1) determination of the age-specific incidence of other agents of diarrhoeal infection in the community (2) a description of risk factors for both *Shigella* as well as other diarrhoeal aetiologies (3) evaluation of seroconversion rate (presence of antibody) to diarrhoeal pathogens at six month intervals in the first five years of life (4) exploration of the relationship between maternal and infant IgG and protection against infection and (5) an investigation into potential host genetic susceptibilities for diarrhoeal disease.

## Methods/Design

**Figure 1:**
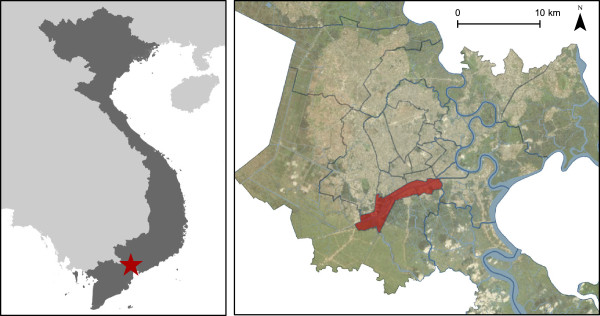
**The location for the cohort study.** Left panel: Location of Vietnam (dark grey) in Southeast Asia, with Ho Chi Minh City shown by the red star. Right panel: A satellite map showing Ho Chi Minh City, with district 8 highlighted in red. The boundaries of other districts are shown in black. Waterways are shown in blue.

In order to achieve our objectives, we have designed a longitudinal cohort study with routine and active surveillance for diarrhoeal disease in district eight of HCHC (Figure 1). This district is a densely populated district in the center of the city with a large number of canals and waterways and is known to contribute proportionally more hospitalized pediatric shigellosis patients than any of the other districts in the city [5].

### Cohort population

Children enrolled into our cohort will be recruited from an existing birth cohort conducted to investigate the incidence of dengue and other viral infections across our study site [24]. Briefly, pregnant mothers living in district eight were enrolled prior to delivery at Hung Vuong Hospital (HVH), a large obstetric hospital in HCMC delivering approximately 44,000 babies annually. Infants were followed up routinely for the first 12 months of life for the purposes of investigating the epidemiology, aetiology and risk factors of a variety of viral infections in infancy. Clinical samples were collected at routine visits corresponding to Expanded Programme on Immunization (EPI) visits at 0, 4, 9 and 12 months of age, including throat and nasopharyngeal swabs as well as a small blood sample. A subset of these children have been followed beyond 12 months of age and are still undergoing routine follow up every six months.

### Enrolment & routine follow up

**Figure 2:**
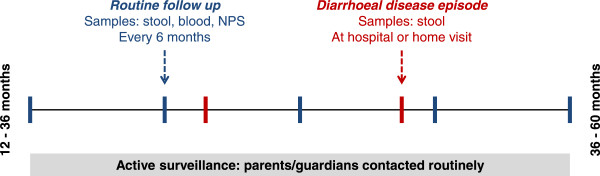
**Flowchart of study design.** Children are enroled at age 12–36 months with routine follow up visits (shown in blue bars) every 6 months. During enrolment and routine follow up visits, a stool sample, blood sample and nasopharyngeal swab (NPS) will be collected in addition to a case report form detailing growth and health information. Follow up will continue for a total of two years for each child, meaning they will exit the cohort at 36–60 months of age. Parents will be contacted on a regular basis to enquire whether their child has experience diarrhoeal disease. When the child experiences an episode of diarrhoea (shown in red bars), study nurses can travel to the home of the participant to a stool sample and a short case report form or the family can bring the child to the Hospital for Tropical Disease for clinical examination. Stool will be collected during diarrhoeal episodes.

Children who had been enrolled in the existing birth cohort will be approached for entry into the diarrhoeal disease cohort between 12–36 months of age at a routine birth cohort follow up visit. An enrolment questionnaire will be administered, detailing information on demographic and socioeconomic characteristics of the household, with a particular focus on water sources, water treatment and toilet practices. Once enrolled, children are requested to attend HVH every six months for a routine health check, as shown in Figure 2. A blood sample (<2 ml), a nasopharyngeal swab and an anal swab or stool sample (if available) is collected by study nurses at these routine visits. Additionally, a questionnaire regarding growth and disease episodes of the child in the preceding six months will be administered. Routine follow up will take place for a total of two years for each child.

### Active & passive case detection

Parents/guardians will be contacted through a text message or phone call on a regular basis to request information on whether the enrolled child has had an episode of diarrhoea in the preceding seven days. Diarrhoea is defined according to the guidelines outlined by the World Health Organization, which is three or more loose stools in a 24 hour period or at least one bloody/mucoid stool [25]. If the parent cannot be reached on the initial attempt, the study nurse will make every effort to establish contact to ensure complete data collection. Once a parent alerts a study nurse to an episode of diarrhoea, the nurse will travel to the participant’s home within a 24-hour window. At the home visit, a stool sample will be collected. If no fresh stool is available at the time of the visit, a sterile stool collection pot will be left with the parents/guardians with instructions for sample collection. Stool pots left at the home will be collected within 24 hours. Furthermore, a short case report form (CRF) will be collected detailing clinical details of the diarrhoeal episode and information on any other known diarrhoeal infections in the household. The parents will also be given oral rehydration packets, probiotics containing *Lactobacillus acidophilus* as well as zinc supplements for the child [26, 27].

If at any point during follow up a parent feels that their child requires medical attention for diarrhoeal disease and feels that a home visit by a nurse is insufficient, they may attend the Hospital for Tropical Disease (HTD) at a subsidised cost (Figure 2). Upon arrival at the hospital the children will be escorted through registration processes by a study nurse who will then lead them to the gastrointestinal ward for clinical evaluation (both inpatient and outpatients). A stool sample will be collected, and a blood sample if clinically indicated. A detailed CRF will be administered collecting clinical, laboratory and haematological information if available.

### Clinical and laboratory investigations

All samples will be labeled with a study number to ensure anonymity and transported to the microbiology laboratory at the Oxford University Clinical Research Unit (OUCRU) for analysis and storage on the same day as collection. Samples from disease episodes will undergo microscopy for blood cells and parasites (*Giardia lamblia*, *Entamoeba histolytica* and *Cryptosporidium*) as well as microbiology culture for *Shigella* spp., *Salmonella* spp., *Campylobacter* spp*.* and *Escherichia coli*. Antimicrobial susceptibility testing of the cultured and identified bacterial pathogens will be performed by the disc diffusion method using the guidelines of the Clinical and Laboratory Standards Institute (CLSI) [28]. Results will be reported back to the treating clinician as soon as they are available.

Aliquots of stool from both healthy routine visits and from disease episodes will be stored at −80°C. Total nucleic acid will be extracted from faecal specimens using the QIAamp viral RNA Mini kit (QIAGEN, Hilden, Germany) or using the Roche MagNA pure 96 automated nucleic acid extraction machine (Roche). RNA will be converted to complementary DNA (cDNA) by reverse transcription (RT) and an aliquot of RNA will be stored −80°C. For RT, extracted RNA will be reverse-transcribed by SuperScript Reverse Transcriptase III and RNAse Inhibitor (Invitrogen) combined with a random hexamer (Roche Diagnostics, UK) according to manufacturer’s instructions. The resulting cDNA will be stored at −80°C. Bacterial PCR will be performed on extracted nucleic acids to potentially increase the diagnosis rate of *Shigella* spp.*, Salmonella* spp. and *Campylobacter* spp*.* Viral pathogens will be identified through batch multiplex realtime PCR procedures to identify rotavirus and norovirus [29]. Additionally, blood samples will be separated into plasma for serology purposes and cells will be stored for future host genetics studies. Specifically, ELISAs will be used to measure IgA, IgM and IgG subtypes to *S. sonnei* O-antigen to evaluate seroconversion rates [30].

### Sample size

We estimate that we will enroll between 650–750 children based on the available population and current attrition rates from the original birth cohort [24]. It is known that the median age of hospitalized *Shigella* infections in HCMC is 24 months [5]. Additionally, the annual incidence of diarrhoea in Vietnam in children under five years of age is 1.5 events/child/year [19]. Therefore, we estimate that we will have 2,100 diarrhoeal episodes in our cohort during two years of follow up. From a cohort study in Hanoi, the *Shigella* positivity rate is approximately 6% of passively detected diarrhoeal cases in children under 5 years [20]. Therefore, we conservatively estimate that we will detect approximately 125 *Shigella* diarrheal episodes over the study period through both passive and active surveillance activities.

### Data management

Each patient will have a unique identifying study code such that sample and documents will not be labeled with any identifying information. Data will be collected electronically whenever possible, including on laptops and on handheld tablets provided to the study nurses and hospital wards. Electronic data entry devices will be password protected and accessible only by authorised users. Paper CRFs will be used in the event of power failure or technical difficulty. A central database has been developed to ensure secure and confidential data management.

### Ethical approval & ethical considerations

Ethical approval for this study has been obtained from the Oxford University Tropical Research Ethics Committee (OxTREC approval 1058–13) as well as from local partners including the Institutional Review Board (IRB) at the Hospital for Tropical Diseases (HTD) and the IRB at Hung Vuong Hospital (HVH). Written informed consent will be obtained from parents/guardians of children at the time of enrolment for both participation as well as storage and future use of pathogenic and human samples. Parents/guardians will be assured that all information generated in this study will remain confidential. Participant families are reimbursed for travel costs to our study sites for routine or disease visits as well as tests involved in the standard of care (full blood count, stool microscopy and microbiology) of diarrhoeal disease at HTD. Furthermore, parents/guardians are free to withdraw their consent for their child at any time and request that study samples not be stored for further testing.

## Discussion

In this work we have described a longitudinal cohort designed to estimate the incidence of diarrhoea due to *Shigella* in the community in HCMC, Vietnam. This study will follow an estimated 650–750 children under the age of five for two years each, collecting information at routine follow up visits and during diarrhoeal disease events through both passive and active surveillance. We aim not only to estimate the incidence of *Shigella* but also to examine the immune response and risk factors for infection and disease.

### Study strengths

The burden of diarrhoeal disease is difficult to accurately estimate [31], and hospitalized illness is generally not representative of infection and disease in the community [32]. Therefore, the most important strength of the study is the active surveillance component as we will be able to estimate the true burden of diarrhoea due not only to S*higella*, but due to a variety of other diarrheal aetiologies in the community as well. Longitudinal blood samples will also provide for the ability to estimate rates seroconversion to *Shigella sonnei* O-antigen [33], which will capture potential *Shigella* infections that we do not identify through our active and passive surveillance. An additional strength of this study is the collaboration with the existing birth cohort established at OUCRU [24]. Such a partnership will allow for data and sample sharing for all participants from birth through at least the first three years of life, providing for the opportunity to explore dynamics of infection and immunity from infancy.

### Limitations

The major, predicted limitations of this study are loss to follow up and incomplete diarrhoeal disease ascertainment. Differential loss to follow up would introduce bias into the results as participants who remain are likely different than those who were lost. Therefore, we will ensure that the rapport between study nurses and participants remains strong through continuous training and maintenance of motivation of the nurses. We have made a significant effort to provide the families with a high level of comprehensive care for diarrhoeal disease in hopes that they will value the overall research programme and continue to participate through the follow up period. Additionally, we are investing heavily in terms of time and personnel to ensure that we capture as many diarrhoeal disease episodes as possible through intensive active surveillance and follow up.

### Future work

The humoral immune response to *Shigella* is partly induced by the O-antigen and is thought to be serotype specific [34, 35]. However, the immune response to the O-antigen as well as other non-polysaccharide *Shigella* antigens in children in endemic areas after symptomatic infection or exposure is extremely ill-defined [36]. We will explore the longitudinal immune response to a range of *Shigella* antigens in infected individuals in an endemic setting with a view of identifying novel vaccine and diagnostic candidates and their serological relationship to the O-antigen. To interrogate the diversity, specificity and longevity of the humoral immune response a range of *Shigella* antigens, a *Shigella* antigen-array will be constructed in collaboration with the Wellcome Trust Sanger Institute. Similar approaches have been used for development of other bacterial vaccines and diagnostics such as *Salmonella* and *Brucella melitensis* [37, 38].

Additionally, we hope to explore the relationship between genetic variation and disease susceptibility through genetic association, candidate gene and genome wide association studies in the future. Host DNA samples will be stored and used for genotyping to investigate potential genetic associations with diarrhoeal disease. Additionally, the development of gastrointestinal and respiratory tract flora will be measured by performing metagenomic analysis from the anal and nasopharyngeal swabs.

## Conclusions

In conclusion, we have designed one of the largest longitudinal, active surveillance cohort studies to study *Shigella* infection and disease in children in Southeast Asia. Through this study, we will be able to estimate an incidence of *Shigella* infection and disease and define the epidemiology of the bacteria in this community, which is representative of many densely crowded, industrializing cities globally. We also hope to eventually more clearly define the longitudinal immune response to this emergent, highly antimicrobial resistant pathogen [12], as well as begin exploration into host susceptibility to infection. Samples, data and analyses from this work are hoped to be a valuable resource to international as well as local medical and public health communities for informing the development and deployment of a *Shigella* vaccine in the future.

## Abbreviations

CLSI: Clinical laboratory standards institute
CRF: Case report form
EPI: Extended programme on immunization
HCMC: Ho Chi Minh City
HTD: Hospital for tropical disease
HVH: Hung Vuong Hospital
NPS: Nasopharyngeal swab
OUCRU: Oxford university clinical research unit
RT: Reverse-transcribed.
